# Exercise Habits, Preferences, Barriers, and Facilitators in Midlife Women

**DOI:** 10.1249/ESM.0000000000000065

**Published:** 2026-05-05

**Authors:** Marnie K. McLean, Angela J. Fong, Jacob M. Haus, Catherine Kim, Lyndsey E. DuBose, Abbi D. Lane

**Affiliations:** 1 School of Kinesiology, University of Michigan, Ann Arbor, MI, USA; 2 Department of Internal Medicine, University of Michigan Medical School, Ann Arbor MI, USA; 3 Department of Health and Exercise Science, Colorado State University, Fort Collins, CO, USA; 4 Department of Medicine, Division of Geriatric Medicine, University of Colorado Anschutz Medical Campus, Aurora, CO, USA

**Keywords:** behaviors, health outcomes, midlife, perimenopause, physical activity, women

## Abstract

**Introduction:**

Menopause usually occurs in midlife and is associated with increased risk of chronic diseases, which can be lowered with exercise. Desired attributes and characteristics of exercise in midlife women are not well-studied. The purpose of this study was to assess exercise habits, preferences, barriers, and facilitators in midlife women.

**Methods:**

In this cross-sectional study, women aged 45–55 yr were invited to participate in an online survey assessing menopause stage and exercise habits, preferences, and interests. Participants were recruited using social media and mass emails sent to employees from a large midwestern university. Data were analyzed using descriptive statistics, Poisson regression, and McNemar’s test. Regression models included age, race, educational attainment, current physical activity, and past physical activity as independent variables.

**Results:**

Eight hundred fifty-seven women who met age screening criteria responded to the survey. Of the 857 total participants, 419 (49.1%) reported regular exercise participation of ≥30 min on most days per week, with most women reporting participation in light aerobic physical activity (668/857; 78.0%); 449 women (52.4%) reported that having a structured exercise program is helpful for participating in exercise. Factors associated with being highly active or active recently included greater physical activity level 1 yr ago (incidence rate ratio: 1.31, 95% confidence interval: 1.28–1.35; *P* = 0.00) and greater educational attainment (incidence rate ratio: 1.02, 95% confidence interval: 1.01–1.05; *P* = 0.004) in adjusted models. Lowering the risk of chronic diseases was rated as an important health outcome. Preferences were not different by menopause status.

**Conclusions:**

Exercise programs could be structured and prioritize time-effectiveness and lowering the risk of chronic disease to appeal to midlife women. These data may inform the development of targeted exercise interventions.

## INTRODUCTION

The benefits of exercise—“structured, planned, and repetitive physical activity aiming to improve or maintain physical fitness” (1)—are well described (2), with a strong dose-response relationship demonstrated between exercise and chronic disease risk reduction (3). The Physical Activity Guidelines for Americans recommend ≥150 min of moderate-to-vigorous aerobic exercise activities and two muscle-strengthening (resistance training [RT]) sessions per week (4). Rates of aerobic exercise and RT participation are lower in women than in men, and overall participation in RT is lower than participation in aerobic exercise among the general population (5). This trend is important because inverse associations between all-cause mortality and physical activity are stronger in women than men (6). Notably, women see greater benefits than men from both lower doses of moderate-to-vigorous aerobic physical activity and muscle-strengthening activities (6).

During the menopause transition (MT), physical activity may decrease while chronic disease risk increases (7–9). Perimenopause is defined as the early stage in MT where menstruation continues yet is irregular. Postmenopause is defined as amenorrhea for 12 consecutive months (10). These stages are characterized by physiological changes for women, including cardiovascular, metabolic, body composition, functional, and musculoskeletal adaptations (9,11). Menopause symptoms, including vasomotor symptoms, vaginal changes, worsened sleep quality, and poor mood (12), may influence activity behaviors. Past research investigating menopause symptoms reported that 354 mostly postmenopause (82%) women wanted to alleviate vasomotor symptoms, poor sleep, memory concerns, and fatigue (13). Estrogen, which decreases but is still available during MT, may be necessary for some cardiovascular adaptations to exercise (14), suggesting perimenopause as a critical intervention point for vascular adaptations (15).

Research indicates that MT influences physical activity participation (16). Ideal strategies for exercise uptake in women in perimenopause are not well described, but midlife Irish women have described social engagement as a motivator to physical activity engagement (16). The purpose of the current study was to investigate behaviors, barriers, facilitators, and preferences regarding exercise in midlife women to better understand how to facilitate exercise early during MT. This information is critical for effectively promoting exercise participation in midlife women. We hypothesized that midlife women would prefer an in-person exercise intervention that incorporates both aerobics and RT since both exercise modalities are known to have health benefits, and participation may be enhanced by social support. We also hypothesized that midlife women would be most interested in exercise programs that can improve vasomotor symptoms, mood, sleep, and fatigue. We aimed to describe exercise behaviors, barriers, facilitators, and preferences in midlife women, further stratified based on demographics, menopause statuses, and self-reported activity levels to design future targeted exercise interventions for midlife women across MT.

## METHODS

### Participants and Procedures

Women aged 45–55 yr were invited to participate in a web-based survey that was developed solely for this project. Digital and printed flyers describing the study and inclusion criteria were distributed, and survey access information was posted on social media, disseminated on mass email lists to staff members of a large midwestern university, and shared with women’s health clinicians. Once participants agreed to complete the survey, they were directed to the survey webpage. Women were included in the study if they met the age criteria of 45–55 yr old; age was manually entered, and those who fell outside the range could not continue completing the survey. Participation was voluntary following viewing of electronic informed consent information. The study was reviewed and deemed exempt by the University of Michigan Institutional Review Board (HUM00256171). Participants were entered into a drawing for $100 virtual gift cards.

### Survey

Survey questions were written and agreed upon by members of the research team based on the relation to physical activity guidelines and extant literature regarding exercise participation in women (Supplemental Content 1, https://links.lww.com/EM9/A53). Responses were collected and managed online using REDCap software (Research Electronic Data Capture; Vanderbilt University, Nashville, TN, USA) (17,18). Participants were queried about their exercise preferences (group size, setting), current habits (type, frequency), perceived barriers and facilitators, and habits 10 yr ago, 1 yr ago, and recently using Likert-scale questions and slider bars (0–100, most important to least important). Participants were asked to describe their physical activity as highly active (>45 min on most days), active (30–45 min on most days), somewhat active (15–30 min on most days), or none. Participants were also provided with open fields to share additional information or expand upon their answers. To ensure that participants were humans and not automated responses (bots), a reCAPTCHA button was used to begin the survey, which is standard of practice (19). To further protect against bots, only responses from individuals who 1) successfully completed the reCAPTCHA and age entry tasks, 2) took at least 3 min to complete the online survey, and 3) moved one of the slider bar responses or filled in a free-text field were included in analysis. Of 933 individuals who entered the survey, 857 met these criteria. No minimum number of responses was required for survey completion. Responses were collected from September to December 2024.

### Statistical Analysis

Survey data were tallied and averaged as frequencies and percentages. McNemar’s test was used to test differences in self-reported exercise participation proportions over time. The associations between current physical activity status and age, race, educational attainment, and past physical activity (1 and 10 yr ago) were assessed using Poisson regression with robust standard errors. Differences in exercise behaviors, preferences, barriers, and facilitators according to menopause status were evaluated using chi-square tests. All analyses were conducted using Stata software 18.0 for Windows (StataCorp LLC, College Station, TX, USA).

## RESULTS

Participant sociodemographic information is presented in Table 1. Women were classified as postmenopause if they answered “Yes” that they had not menstruated in the last 12 months. Women were classified as in perimenopause if they answered “No” or “Not Sure” that they had not menstruated in the last 12 months and reported menstrual irregularities in the last 6 months. Women were classified as premenopause if they answered “No” or “Not Sure” that they had not menstruated in the last 12 months and did not report menstrual irregularities in the last 6 months. Women were classified as unknown if they did not meet the above classifications.

**Table 1:** Participant Demographics and Activity Statuses (*n* = 857).

Characteristic	
Age (yr), mean ± standard deviation	50.46 ± 3.13
Race, *n* (%)	
Asian	39 (4.6)
Hispanic or Latino/a/e	26 (3.0)
Native American or First Nation	5 (0.6)
Native Hawaiian or Pacific Islander	2 (0.2)
White	740 (86.4)
Black	60 (7.0)
Other^*a*^	8 (0.9)
Education, *n* (%)	
Did not finish high school	1 (0.1)
High school diploma	14 (1.6)
Some college	84 (9.9)
Associate’s degree	55 (6.5)
Bachelor’s degree	318 (37.3)
Master’s degree	293 (34.4)
Doctoral or professional degree	88 (10.3)
Current physical activity status^*b*^, *n* (%)	
Highly active	135 (15.8)
Active	284 (33.3)
Somewhat active	312 (36.5)
None	123 (14.4)
Physical activity status 1 yr ago^*b*^, *n* (%)	
Highly active	116 (13.6)
Active	294 (34.5)
Somewhat active	350 (41.2)
None	92 (10.8)
Physical activity status 10 yr ago^*b*^, *n* (%)	
Highly active	201 (23.5)
Active	364 (42.6)
Somewhat active	237 (27.8)
None	52 (6.1)
Marital status, *n* (%)	
Married	597 (70.2)
Single	79 (9.3)
Widowed	11 (1.3)
In a relationship	67 (7.9)
Divorced	88 (10.3)
Separated	9 (1.1)
Employment status, *n* (%)	
Full-time outside the home	770 (90.2)
Working inside the home/caretaking	18 (2.1)
Part-time outside the home	45 (5.3)
Retired	2 (0.2)
Unemployed	1 (0.1)
Other^*c*^	18 (2.1)
Menopause status, *n* (%)	
Premenopause	114 (13.3)
Perimenopause	312 (36.4)
Postmenopause	297 (34.8)
Unknown	134 (15.6)

Not all percentages add up to 100% based on questions left unanswered by survey respondents.

^*a*^Other race includes Middle Eastern identities, mixed races, religious ethnicity.

^*b*^Physical activity status was defined as follows: none; somewhat active, 15–30 min on most days; active, 30–45 min on most days; highly active, >45 min on most days.

^*c*^Other employment includes multiple jobs, hybrid/remote workplaces, and medical leave.

Significantly more women reported being active or highly active 10 yr ago (66.1%) versus 1 yr ago or recently (49.1%; *P* > 0.001). There was no significant difference in the proportion of women reporting being highly active/active recently versus 1 yr ago (*P* = 0.56). In our adjusted regression model including age, race, and physical activity 10 yr ago, physical activity 1 yr ago was associated with a higher prevalence of active or highly active current physical activity level (incidence rate ratio: 1.31, 95% confidence interval: 1.28–1.35; *P* = 0.00). Educational attainment was also significantly associated with active or highly active current physical activity (incidence rate ratio: 1.02, 95% confidence interval: 1.01–1.05; *P* < 0.01).

**Figure 1.**
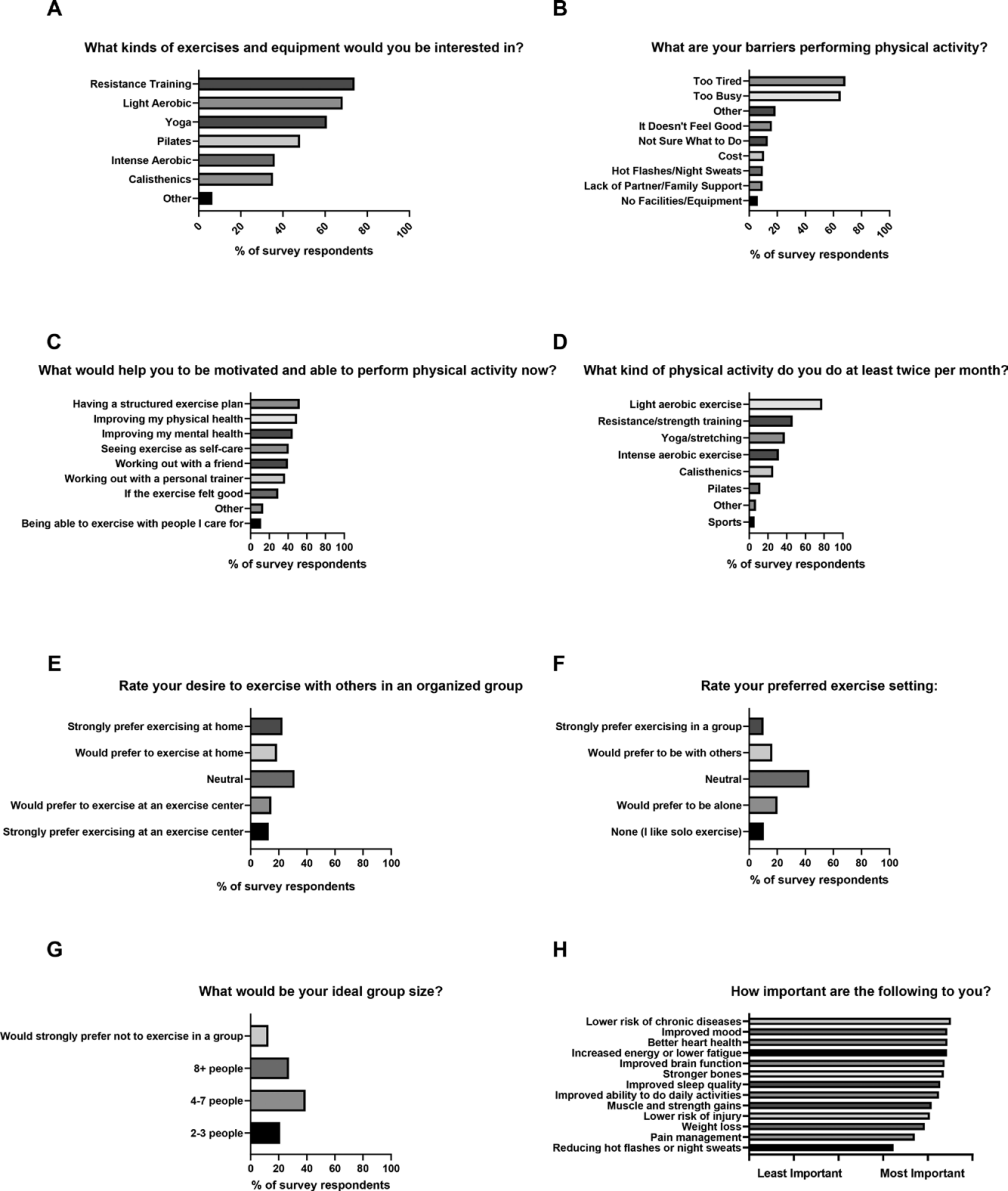
Survey responses regarding exercise habits, preferences, barriers, and facilitators in midlife women. A–G. Survey questions shown with percentages of respondents selecting each answer. H. Survey question shown with ratings of importance for each outcome. Light aerobic exercise (A, D) examples in the survey included walking, light jogging, low impact aerobics, leisurely cycling, or swimming. Intense aerobic exercise (A, D) examples included running, fast cycling or swimming, and high intensity interval training. Resistance/strength training (A, D) examples included free weights, resistance bands, weight machines, and bodyweight exercises. Calisthenics (A, D) examples included pushups, pull-ups, sit-ups, and body weight squats.

Questions and responses regarding exercise preferences, habits, barriers, and facilitators are shown in Figure 1. The most frequently selected exercise modality of interest was RT (free weights, resistance bands, weight machines, bodyweight exercises), which was selected by 74.0% (634/857) of respondents (Fig. 1). Modality preferences were not different by menopause status (*P* = 0.36). The most frequently selected barriers to physical activity were being too tired (587/857; 68.5%) and too busy (558/857; 65.1%) (Fig. 1). Barriers were not different by menopause status (*P* = 0.29). The most frequently selected facilitators of physical activity were having a structured exercise plan (449/857; 52.4%), improving physical health (425/857; 49.6%), and improving mental health (385/857; 44.9%) (Fig. 1). Motivators were not different by menopause status (*P* = 0.38). Most women (668/857; 78.0%) reported participating in light physical activity (walking, light jogging, low impact aerobics, leisurely cycling, or swimming) (Fig. 1). Participants reported mostly neutral preferences on home- or center-based exercise training and exercising alone versus in a group setting (Fig. 1). When asked about the ideal group size for exercise, most women selected 4–7 group members (Fig. 1). Setting and group preferences did not differ by menopause status (*P* > 0.05). On a series of questions asking participants to rate various facets of potential health benefits from exercise on a scale from very important to not important at all, lowered risk of chronic diseases, improved heart health, and improved mood were rated as most important (Fig. 1). Vasomotor symptom reduction was rated as the least important (Fig. 1).

In women who reported no (*n* = 123) or some (*n* = 312) physical activity recently, roughly equivalent to not meeting national physical activity guidelines, being too tired was the most frequently selected barrier to physical activity; in women who reported being active (*n* = 284) or highly active (*n* = 135), being too busy was the most frequently selected barrier (table, Supplemental Content 2, https://links.lww.com/EM9/A54). The most frequently selected facilitator or motivator of physical activity was having a structured exercise plan for women reporting no, some, and high physical activity; women reporting being active selected improving their physical health most frequently (Supplemental Table, Supplemental Content 3, https://links.lww.com/EM9/A55). Women completing no physical activity most often selected interest in light physical activity, whereas women reporting being somewhat active, active, or highly active most often selected interest in RT (Supplemental Table, Supplemental Content 4, https://links.lww.com/EM9/A56). Women completing no physical activity rated improving energy levels as most important, whereas women who reported being somewhat active, active, or highly active rated lowering chronic disease risk as most important (Supplemental Figure, Supplemental Content 5, https://links.lww.com/EM9/A57).

## DISCUSSION

This study provides valuable information from a large group of midlife women regarding their exercise behaviors, preferences, barriers, and facilitators. The major findings of this study include 1) current physical activity participation was associated with physical activity levels 1 yr ago and educational attainment; 2) midlife women most frequently selected RT as their modality of interest and lowering their risk of chronic disease, improving their mood, and improving heart health as benefits of exercise that were important to them; and 3) midlife women were neutral on center- versus home-based physical activity and exercising alone versus in a group setting. Most findings were consistent regardless of menopause status.

The reasons for lower physical activity and desired exercise program characteristics are not well described in midlife women (20), yet long-term exercise participation is crucial for its health benefits (21,22). Physical activity 1 yr ago and educational attainment were associated with higher likelihood of current physical activity in our sample. The positive association between past physical activity and current physical activity has been previously described (23). Interestingly, physical activity 10 yr ago was not a significant predictor of current physical activity participation in our study. Physical activity levels tend to decrease throughout the lifespan (24), including during MT (7,8). Thus, responses about physical activity 1 yr ago may have better captured levels during a similar life phase for midlife women. Educational attainment has been associated with health outcomes and health behaviors, with higher educational attainment being related to higher physical activity levels in adulthood (25), which our research in midlife women supports.

In a prior study, midlife women reported health concerns as both a barrier to and a facilitator of physical activity participation (26). Although vasomotor symptoms may not be improved by exercise (27), other menopause symptoms appear to be improved (28). Vasomotor symptom management rated as least important compared to other health outcomes in our study. Midlife women have previously reported preferences for interventions that improve vasomotor symptoms, sleep, concentration, and fatigue (13). Other research has indicated that management of menopause symptoms, future health status, social engagement, and role models were motivational reasons to increase physical activity participation (16). Our research does not align with these findings; however, our survey did not ask about women’s vasomotor symptoms, so we cannot identify individuals affected by vasomotor symptoms in our study.

Our study reports similar barriers and facilitators as a prior study of women who completed focus groups on adherence to weight loss practice (29). In that study, women stated that lack of time and stress inhibited adherence, whereas lowering the risk of chronic disease was a major motivator for losing weight (29). Exercise interventions should take into consideration which exercise programming can address these health outcomes. A study examining adherence to exercise in midlife women suggested that facilitators of increasing exercise adherence in midlife were structured activities as part of a routine, positive feelings related to physical activity, and feeling accountable to others (30). The barriers reported in the study were competing demands and disruptions to daily schedules (30), which is supported by our finding that women reported being too busy and too tired as their greatest barriers. Time is often reported as a barrier to meeting exercise guidelines (31–34). In a study of predominantly women in perimenopause, participants reported that barriers to weight loss included menstrual changes, somatic pain, caretaking responsibilities, and lack of time and motivation (29). Programs that provide instruction and safe places for participants to exercise are recommended to promote uptake and adherence (35). This, coupled with our finding that most women reported that a structured exercise program would motivate them to partake in exercise, is an important consideration for midlife women.

Responses to our study indicated that women were neutral on center- versus home-based physical activity and exercising alone versus in a group setting. Another study found that women prefer face-to-face, one-on-one exercise with a personal trainer (36). In the same study, which queried women (35% premenopause, 14% perimenopause, 39% postmenopause, and 12% unknown status) about their exercise preferences, 97% reported wanting a walking intervention, followed by dancing (75%) and swimming (74%); importantly, RT was not included as an exercise modality option in the study (36). Overall, RT was the most selected modality of interest in our study, except in women reporting no physical activity. This finding may be related to prior research investigating why women of all ages tend to report lower rates of participation in RT (37). That integrative review detailed different barriers to participation reported by women, including time constraints, knowledge and education, modality and intensity, social support, and behavioral strategies (37).

### Limitations

Our study has a few limitations. Most survey responses followed a mass email sent to university staff, indicating that university employees made up a large portion of respondents; therefore, our study may not fully capture the understanding of women-identifying persons’ exercise preferences or needs. Most respondents were working full-time outside the home and had at least a bachelor’s degree. These women may have different responsibilities and time constraints than those with different occupations or living in different geographical locations. Our survey only queried women between 45 and 55 yr of age, which was chosen to capture women in perimenopause. Expansion of the age criteria would have encompassed midlife women more broadly, including those experiencing early or late MT. Approximately half of our survey respondents reported being active or highly active (roughly corresponding to meeting or exceeding guidelines for physical activity), which is much higher than the 19% of American women reporting meeting aerobic and RT components of the national physical activity guidelines (4). Thus, our findings might not extend to other populations. The survey used has not been validated, potentially introducing researcher bias through survey development and interpretation. The survey did not query participants regarding exercise participation for improved physical appearance, which may also be a facilitator of participation. Finally, participants’ menopause statuses may have been misclassified since the survey instrument did not include a question regarding history of hysterectomy or oophorectomy.

### Conclusion

Women who participated in physical activity 1 yr ago and women with higher educational attainment were more likely to report current physical activity at levels similar to national physical activity guidelines. Multiple preferences, barriers, motivators, and facilitators of exercise engagement in midlife women were defined. This study contributes to existing literature regarding exercise uptake in midlife women by providing information that can be incorporated and addressed when designing exercise programs and interventions. When designing exercise interventions for midlife women, researchers should consider prioritizing programs designed to reduce chronic disease risk, improve cardiovascular health, and improve mood. Interventions could include RT and consider time-efficient and nonexhausting routines to address the barriers of being too busy or too tired to exercise.

## ACKNOWLEDGMENTS

We thank the University of Michigan grant (UM1TR004404) for providing access to REDCap services. The results of the study are presented clearly, honestly, and without fabrication, falsification, or inappropriate data manipulation. The results of the present study do not constitute endorsement by the American College of Sports Medicine.

## CONFLICTS OF INTEREST AND SOURCE OF FUNDING

None of the authors have any conflicts of interest to report. M.K.M. received a Rackham Graduate Student Research Grant. Access to REDCap services was provided by a University of Michigan grant (UM1TR004404).

## DATA AVAILABILITY

All data generated and/or analyzed during the current study are included in the published article and its supplemental content.

## Supplementary Material
