# Supplementary material for: Exercise Habits, Preferences, Barriers, and Facilitators in Midlife Women
Source: Exerc Sport Mov. 2026 May 5;4(3):e00065. doi: 10.1249/ESM.0000000000000065 (PMC13143370; doi:10.1249/ESM.0000000000000065)
Supplement: Supplementary file 1 [file esam-4-e00065-s001.pdf]

Supplemental Content 1. Survey.

Survey Questions:

First, we'd like to know a little bit about you:

1. How old are you?
    a. 45-55 years old [eligible, continue from here]
2. What is your race and ethnic background? (Select all that apply)
    a. Black or African American
    b. Asian
    c. Hispanic or Latino
    d. Native American or First Nation People
    e. Native Hawaiian or Pacific Islander
    f. White
    g. Unknown
    h. Other (please describe) ___________
3. What is your highest level of education?
    a. Did not finish high school
    b. High school
    c. Some college
    d. Associate's degree
    e. Bachelor's degree
    f. Master's degree
    g. Doctoral or Professional degree
4. What is your marital status?
    a. Married
    b. In a relationship
    c. Widowed
    d. Divorced
    e. Separated
    f. Single
5. What is your employment status?

a. Full-time outside the home
b. Working inside the home/caretaking
c. Part-time outside the home
d. Student
e. Retired
f. Unemployed
g. Unable to work
h. Other (please describe) _______

Physical activity is any movement of your body. It can include walking, running, dancing, lifting weights, swimming, exercise classes, or sports. Questions in this survey refer to <u>any</u> of these types of activities.

1. What was your physical activity level like **10 years ago**?
    a. None
    b. Somewhat (15-30 minutes on most days)
    c. Active (30-45 minutes on most days)
    d. Highly active (>45 minutes on most days)
2. What was your physical activity level like **1 year ago**?
    a. None
    b. Somewhat (15-30 minutes on most days)
    c. Active (30-45 minutes on most days)
    d. Highly active (>45 minutes on most days)
3. What has your physical activity level been like **recently (in the last month)?**
    a. None
    b. Somewhat (15-30 minutes on most days)
    c. Active (30-45 minutes on most days)
    d. Highly active (>45 minutes on most days)
4. If you reported being Active or Highly Active **recently**, can you share what helped you achieve that?
    a. ____________
5. What are your barriers performing physical activity? (Select all that apply, even if you are physically active)

a. Lack of partner/family support
b. No facilities/equipment
c. Not sure what to do
d. Too tired
e. Too busy
f. It doesn't feel good
g. Cost
h. Hot flashes/night sweats
i. Other (please describe) ____________________________

6. What would help you to be motivated and able to perform physical activity now? (Select all that apply)
   a. Working out with a friend
   b. Working out with a personal trainer
   c. Having a structured exercise plan
   d. Improving my physical health
   e. Improving my mental health
   f. If the exercise felt good
   g. Being able to exercise with people I care for
   h. Seeing exercise as self-care
7. If you are active now, what kind of physical activity do you do at least twice per month? (Select all that apply)
   a. Light aerobic exercise (walking, light jogging, low impact aerobics, leisurely cycling or swimming)
   b. Intense aerobic exercise (running, fast cycling or swimming, HIIT, etc.)
   c. Resistance/strength training (free weights, resistance bands, weight machines, bodyweight exercises)
   d. Yoga/stretching
   e. Pilates
   f. Calisthenics (push-ups, pull-ups, sit-ups, body weight squats, etc.)
   g. Sports (please describe) ________
   h. Other (please describe) _________

8. What kinds of exercises and equipment would you be interested in? (select all that apply)
    a. Light aerobic exercise (walking, light jogging, low impact aerobics, leisurely cycling or swimming, etc)
    b. Intense aerobic exercise (running, rowing, fast cycling or swimming, HIIT, etc.)
    c. Resistance/strength training (free weights, resistance bands, weight machines, bodyweight exercises)
    d. Yoga
    e. Pilates
    f. Calisthenics (push-ups, pull-ups, sit-ups, body weight squats, etc.)
    g. Other _________
9. Rate your preferred exercise setting:
    a. Strongly prefer exercising at home
    b. Would prefer to exercise at home
    c. Neutral
    d. Would prefer to exercise at an exercise center
    e. Strongly prefer exercising at an exercise center
10. Please rank which benefits of exercise training are most important to you personally (1 being most important). **Each option was linked to a slider bar from which a 1-5 scale was used to rate the option from Most (1) to Least (5) Important; lower numbers indicate greater Importance**
    a. Muscle or strength gains
    b. Better heart health
    c. Stronger bones
    d. Lower risk of chronic diseases, like diabetes and cancer
    e. Improved mood
    f. Weight loss
    g. Improved ability to do daily activities
    h. Improved brain function
    i. Increased energy/lower fatigue
    j. Reduce hot flashes and night sweats

k. Pain management
l. Lower risk of injury
m. Improved sleep quality
n. Other: _______________________

11. Rate your desire to exercise with others in an organized group
    a. None (I like solo exercise)
    b. Would prefer to be alone
    c. Neutral
    d. Would prefer to be with others
    e. Strongly prefer exercising in a group
12. If you were to exercise in a group setting, what would be your ideal group size?
    a. 2-3 people
    b. 4-7 people
    c. 8+ people
    d. Would strongly prefer not to exercise in a group
13. Which of the following settings would best motivate you to maintain a consistent resistance training schedule?
    a. Exercise at home at your own pace and your own schedule with video guidance
    b. Exercise at home on a schedule with group training sessions over Zoom
    c. A mix of mostly home exercise with occasional group sessions at a fitness gym led or guided by a personal trainer
    d. A mix of home exercise and frequent group sessions at a fitness gym led or guided by a personal trainer
    e. Always exercising in a group at a fitness gym led or guided by a personal trainer
14. Have you gone through menopause (no menstrual cycle for longer than 12 months)?
    a. Yes
    b. No
    c. Not sure
15. If no, have you experienced any irregularities in your menstrual cycle in the past 6 months?
    a. Yes

b. No

c. Not sure

16. Is there any other information you would like to share about your feelings about exercise and menopause?

a. ____
