# Supplementary material for: Exercise Habits, Preferences, Barriers, and Facilitators in Midlife Women
Source: Exerc Sport Mov. 2026 May 5;4(3):e00065. doi: 10.1249/ESM.0000000000000065 (PMC13143370; doi:10.1249/ESM.0000000000000065)
Supplement: Supplementary file 2 [file esam-4-e00065-s002.pdf]

Supplemental Content 2. Barriers to physical activity (PA) by PA level.

**Barrier**	**No PA (*n*=123)**	**Some PA (*n*=312)**	**Active (*n*=284)**	**Highly Active (*n*=135)**
**Lack of support**	17 (13.82%)	37 (11.86%)	19 (6.69%)	8 (5.93%)
**Lack of facilities**	15 (12.20%)	19 (6.09%)	14 (4.93%)	4 (2.96%)
**Not sure what to do**	28 (22.76%)	46 (14.74%)	30 (10.56%)	9 (6.67%)
**Too tired**	*106 (86.18%)*	*227 (72.76%)*	182 (64.08%)	71 (52.59%)
**Too busy**	76 (61.79%)	201 (64.42%)	*197 (69.37%)*	*82 (60.74%)*
**It feels bad**	19 (15.45%)	70 (22.44%)	40 (14.08%)	9 (6.67%)
**Cost**	13 (10.57%)	37 (11.86%)	28 (9.86%)	11 (8.15%)
**Vasomotor symptoms**	20 (16.26%)	45 (14.42%)	13 (4.58%)	4 (2.96%)
**Other**	30 (24.39%)	61 (19.55%)	42 (14.79%)	27 (20.00%)

PA status defined as follows: none; somewhat active, 15-30 min on most days; active, 30-45 min on most days; highly active, >45 min on most days. Other barriers included pain, urinary incontinence, boredom, and medical diagnoses. Italicized values indicate most selected barriers.
