# Supplementary material for: Exercise Habits, Preferences, Barriers, and Facilitators in Midlife Women
Source: Exerc Sport Mov. 2026 May 5;4(3):e00065. doi: 10.1249/ESM.0000000000000065 (PMC13143370; doi:10.1249/ESM.0000000000000065)
Supplement: Supplementary file 3 [file esam-4-e00065-s003.pdf]

Supplemental Content 3. Facilitators of physical activity (PA) by PA level.

Facilitator	No PA (*n*=123)	Some PA (*n*=312)	Active (*n*=284)	Highly Active (*n*=135)
**Working out with a friend**	50 (40.65%)	124 (39.74%)	115 (40.49%)	51 (37.78%)
**Having a personal trainer**	41 (33.33%)	120 (38.46%)	111 (39.08%)	43 (31.85%)
**Having a structured exercise plan**	*74 (60.16%)*	*171 (54.81%)*	136 (47.89%)	*68 (50.37%)*
**Improving my physical health**	63 (51.22%)	147 (47.12%)	*147 (51.76%)*	67 (49.63%)
**Improving my mental health**	59 (47.97%)	123 (39.42%)	137 (48.24%)	65 (48.15%)
**If it feels good**	37 (30.08%)	87 (27.88%)	92 (32.39%)	37 (27.41%)
**Being able to exercise with those I care for**	12 (9.76%)	28 (8.97%)	37 (13.03%)	20 (14.81%)
**Seeing exercise as self-care**	50 (40.65%)	109 (34.94%)	128 (45.07%)	61 (45.19%)
**Other**	22 (17.89%)	45 (14.42%)	32 (11.27%)	16 (11.85%)

PA status defined as follows: none; somewhat active, 15-30 min on most days; active, 30-45 min on most days; highly active, >45 min on most days. Other facilitators included more time, designated space at home, and sleep improvements. Italicized values indicate most selected facilitators.
