# Supplementary material for: Exercise Habits, Preferences, Barriers, and Facilitators in Midlife Women
Source: Exerc Sport Mov. 2026 May 5;4(3):e00065. doi: 10.1249/ESM.0000000000000065 (PMC13143370; doi:10.1249/ESM.0000000000000065)
Supplement: Supplementary file 4 [file esam-4-e00065-s004.pdf]

Supplemental Content 4. Physical activity (PA) modality interest by PA level.

Interest	No PA (*n*=123)	Some PA (*n*=312)	Active (*n*=284)	Highly Active (*n*=135)
**Light aerobic exercise**	*96 (78.05%)*	216 (69.23%)	186 (65.49%)	87 (64.44%)
**HIIT exercise**	23 (18.70%)	80 (25.64%)	126 (44.37%)	80 (59.26%)
**Resistance/strength training**	80 (65.04%)	*225 (72.12%)*	*220 (77.46%)*	*107 (79.26%)*
**Yoga**	78 (63.41%)	185 (59.29%)	182 (64.08%)	74 (54.81%)
**Pilates**	55 (44.72%)	140 (44.87%)	149 (52.46%)	67 (49.63%)
**Calisthenics**	39 (31.71%)	86 (27.56%)	113 (39.79%)	64 (47.41%)
**Other**	9 (7.32%)	16 (5.13%)	21 (7.39%)	10 (7.41%)

PA status defined as follows: none; somewhat active, 15-30 min on most days; active, 30-45 min on most days; highly active, >45 min on most days. HIIT, high intensity interval training. Other options included sports and dance. Italicized values indicate the most selected modalities.
