# Supplementary material for: Exercise Habits, Preferences, Barriers, and Facilitators in Midlife Women
Source: Exerc Sport Mov. 2026 May 5;4(3):e00065. doi: 10.1249/ESM.0000000000000065 (PMC13143370; doi:10.1249/ESM.0000000000000065)
Supplement: Supplementary file 5 [file esam-4-e00065-s005.pdf]

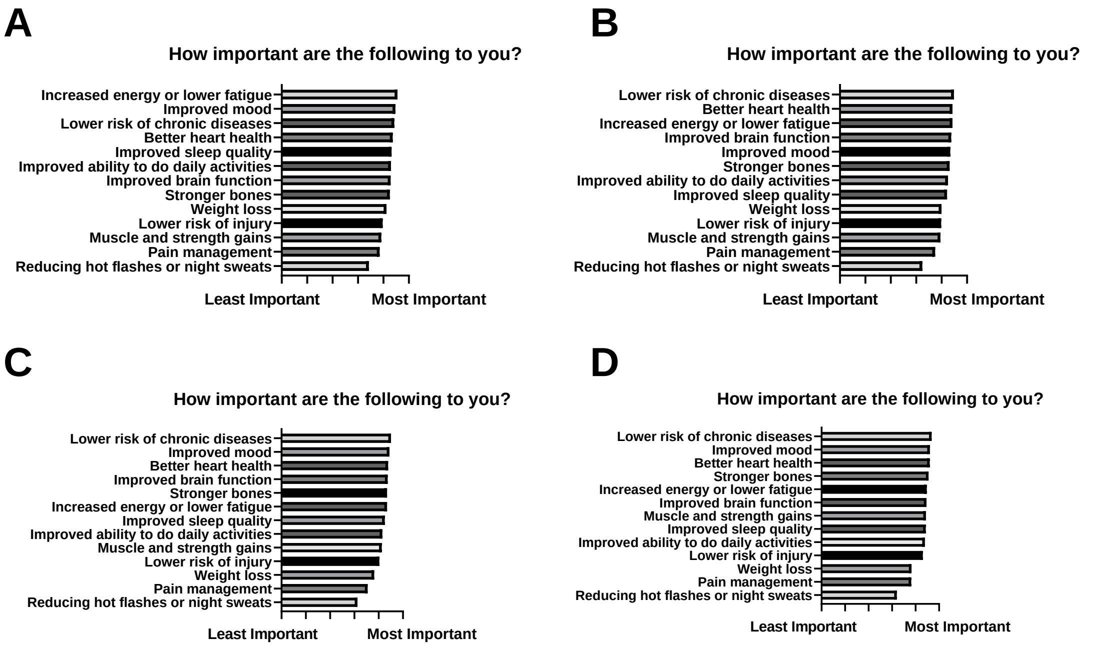

Supplemental Content 5. Exercise outcome interest by physical activity level. A. Not physically active. B. Somewhat active: 15-30 min on most days. C. Active: 30-45 min on most days. D. Highly active: >45 min on most days. The rating of importance for each outcomes is shown.
